# A biologically based mathematical model for spontaneous and ionizing radiation cataractogenesis

**DOI:** 10.1371/journal.pone.0221579

**Published:** 2019-08-23

**Authors:** Tetsuya Sakashita, Tatsuhiko Sato, Nobuyuki Hamada

**Affiliations:** 1 Department of Radiation-Applied Biology Research, Quantum Beam Science Research Directorate, National Institutes for Quantum and Radiological Science and Technology (QST), Watanuki, Takasaki, Gunma, Japan; 2 Research Group for Radiation Transport Analysis, Nuclear Science and Engineering Center, Japan Atomic Energy Agency (JAEA), Shirakata, Tokai, Ibaraki, Japan; 3 Radiation Safety Research Center, Nuclear Technology Research Laboratory, Central Research Institute of Electric Power Industry (CRIEPI), Iwado-kita, Komae, Tokyo, Japan; ENEA Centro Ricerche Casaccia, ITALY

## Abstract

Cataracts have long been known, but a biomathematical model is still unavailable for cataratogenesis. There has been a renewed interest in ionizing radiation cataracts because the recent international recommendation of the reduced lens dose limit stimulated the discussion toward its regulatory implementation in various countries. Nevertheless, a relationship between radiation (dose and dose rate) and response (e.g., incidence, onset and progression) remains incompletely understood, raising the need for a risk-predictive mathematical model. We here report for the first time an *in silico* model for cataractogenesis. First, a simplified cell proliferation model was developed for human lens growth based on stem and progenitor cell proliferation as well as epithelial-fiber cell differentiation. Then, a model for spontaneous cataractogenesis was developed to reproduce the human data on a relationship between age and cataract incidence. Finally, a model for radiation cataractogenesis was developed that can reproduce the human data on a relationship between dose and cataract onset at various ages, which was further applied to estimate cataract incidence following chronic lifetime exposure. The model can serve as the foundation for further development of the risk-predictive model for cataractogenesis along with additional considerations of various biological mechanisms and epidemiological datasets.

## Introduction

Nearly a millennium has passed since the term “cataract” was coined [1]. A cataract is a clouding or opacity of the normally transparent lens of the eye, and is the leading cause of visual impairment and blindness worldwide [2]. Age-related (senile) cataracts are most common, and proven cataractogenic factors include ionizing radiation (IR), ultraviolet (UV) radiation, corticosteroids, diabetes, obesity and cigarette smoking [3–5]. The three major types of cataracts classified according to its anatomical location are posterior subcapsular (PSC), cortical, and nuclear. Of these, cortical and PSC types are most common in senile and IR cataracts, respectively [6,7].

Soon after discovery of X-rays in 1895, the first report on IR cataracts in animals (rabbits) appeared in 1897 [8], followed by that in humans in 1903 [9]. Nonetheless, a surge of interest in radiation protection of the ocular lens was generated by the observation of cataracts in atomic bomb survivors and cyclotron workers in the late 1940s [4,5], both studies suggesting that with sufficiently high dose, cataracts could be a fairly rapid sequela of radiation exposure. The International Commission on Radiological Protection (ICRP) has set lens dose limits since 1954 [10], and recommended its significant reduction in 2011 leading to a resurgence of interest radiation protection of the lens [11]. Currently, ICRP assumes that cataracts are tissue reactions (formerly called nonstochastic or deterministic effects) with a dose threshold, there is no dose rate effect, and all minor opacities progress to vision impairing cataracts [11]. These assumptions, however, need further scientific validation as recently recommended by the US National Council on Radiation Protection and Measurements (NCRP) [1], because manifestations of IR cataracts (e.g., the shape of the dose response curve, the dose and dose rate dependence of the temporal kinetics for onset and progression) and its underpinning mechanisms remain incompletely understood [12,13].

Both ICRP and NCRP have recently emphasized the need of a more mechanistically based model for radiation protection purposes [14,15]. Besides, the need of integrating biology and epidemiology for radiation cataracts has recently been highlighted [16]. However, mathematical modeling of the lens growth, spontaneous cataractogenesis and IR cataractogenesis has not been described in the literature, with a single exception of one report on cell-density distribution in the lens epithelium of animal lenses [17]. As the first attempt, the current study was undertaken to develop a simplified cell population model for human lens growth, and an *in silico* model for spontaneous and IR cataractogenesis that can reproduce the human cataract data.

## Development of a simplified cell population model for human lens growth

**Fig 1:**
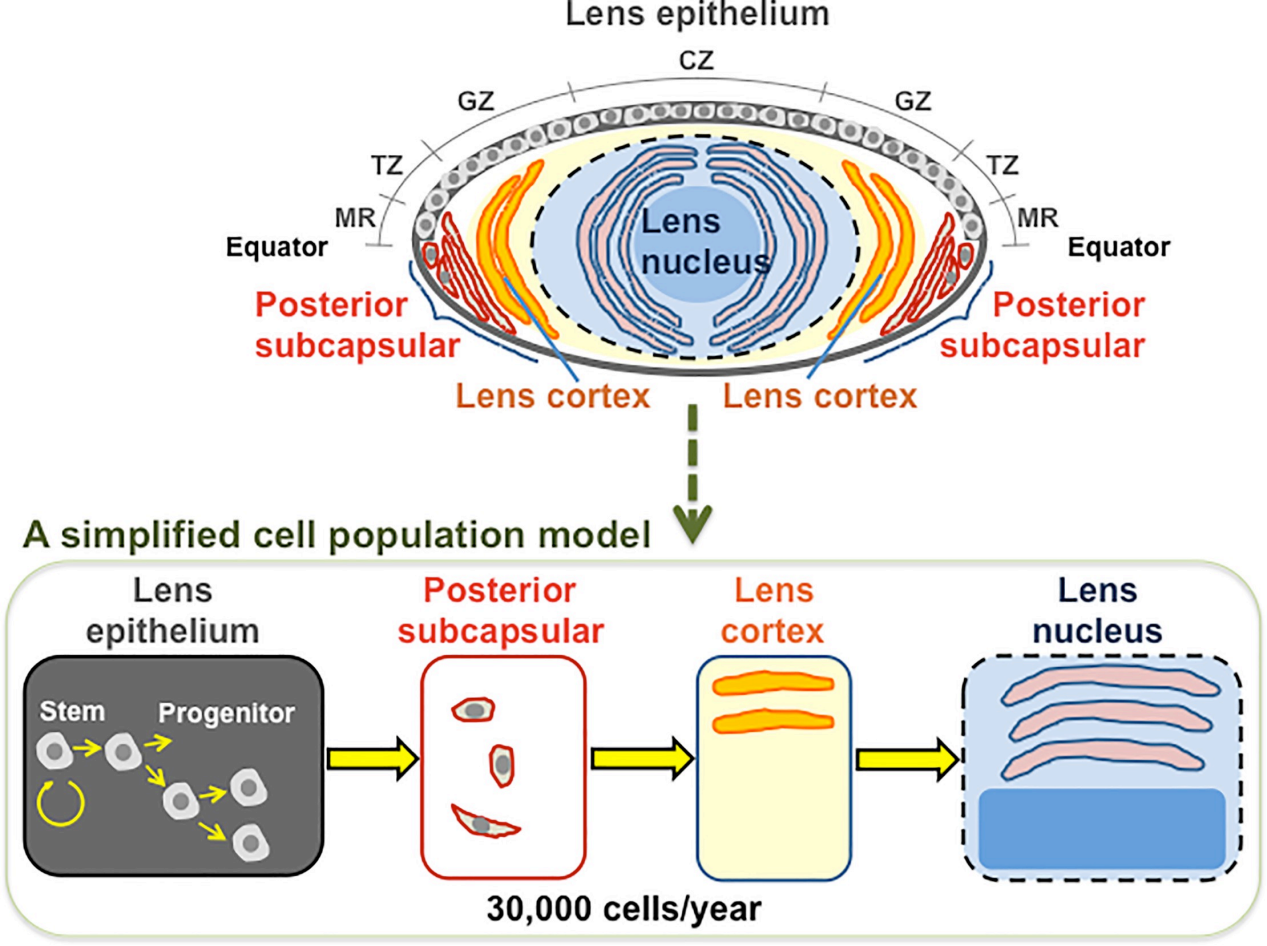
A simplified cell population model. The lens epithelium consists of a single layer of lens epithelial cells (LECs) located in the anterior subcapsular region. The central zone (CZ), germinative zone (GZ), transitional zone (TZ) and meridional rows (MR) compose the lens epithelium. LECs in the GZ around the equator divide, migrate posteriorly, and differentiate into elongated lens fiber cells (LFCs). Newly formed LFCs surround existing cortical LFCs and become more internalized, culminating in production of highly ordered, tightly packed mature nuclear LFCs. These processes are simplified here, where cell migration is assumed to occur once a year.

We first simulate human lens growth by modeling each cell in the lens, where each cell in the lens surface grows and migrates to the lens core with simple cell population dynamics (the structure of the lens depicted in Fig 1).

The lens capsule, the lens epithelium and growth shells constitute the lens, and the boundary between its anterior and posterior surfaces is called an equator. The lens capsule is an outermost smooth basement membrane that encases the lens. The lens epithelium is a simple cuboidal epithelium in the anterior subcapsular region, and consists of the central zone (CZ), germinative zone (GZ), transitional zone (TZ) and meridional rows (MR). Lens epithelial cells (LECs) in the GZ divide, differentiate around the equator to produce lens fiber cells (LFCs) devoid of cellular organelles, and migrate posteriorly. Lens nucleus and lens cortex compose growth shells. Lens growth continues throughout life, during which time nascent LFCs form a new growth shell exterior to the underlying, older LFCs and become more internalized and tightly packed. Primary LFCs comprise the embryonic lens nucleus (formed in the first 6 weeks after fertilization), and secondary LFCs comprise the fetal lens nucleus (formed from the seventh week until birth), the juvenile lens nucleus (formed from birth until puberty), the adult lens nucleus and the cortex (formed from puberty onwards). LFCs newly formed after birth are thus added to juvenile and adult lens nuclei.

The exponential growth phase of the human lens begins after conception, followed by a linear growth phase from around age 3 years onwards [18]. The human data of François [19] were well fitted at age 1–90 years by the following linear model:

$$lens\;volume\;({mm}^{3})=1.205\times age\;(in\;years)+134.05$$ (1)

from which the growth rate can be determined as 1.205 mm^3^/year throughout life (Table 1). An increase in the lens volume was assumed to result from an increase in the number of a single cell type (i.e., LFCs). The mean cross-sections of LFCs at juvenile and adult lens nuclei were reported as 14 and 7 μm^2^, respectively [20]: its arithmetic mean (i.e., 10.5 μm^2^) was assumed for all LFCs (Table 1). Young LFCs are uniform in shape and can be as long as 8–10 mm, but middle-aged LFCs are less uniform [21]: the length of all LFCs was therefore set as 4 mm (Table 1). With these numbers, the volume was set as 4.2 × 10^−5^ mm^3^/cell (calculated as the mean cross-section of 10.5 μm^2^ × the mean length of 4 mm) for all LFCs, and the growth rate was hence set as 3 × 10^4^ cells/year [= 1.205 mm^3^/year/4.2 × 10^−5^ mm^3^/cell = 2.869 × 10^4^ cells/year ≈ 3 × 10^4^ cells/year (rounded up)] (Table 1). All LFCs that accumulate at 3 × 10^4^ cells/year in juvenile and adult lens nuclei should come from the PSC region or the lens cortex, but LFCs in the PSC region or the lens cortex do not divide. Therefore, 3 × 10^4^ cells were assumed to move from the lens epithelium (where dividing cells exist) to the PSC region, then the lens cortex, and finally the juvenile or adult lens nucleus every year (Fig 1).

**Table 1:** Parameters used in a cell population model.

Mean growth rate (mm^3^/year)	1.205
Mean cross-section of lens fiber cell (μm^2^)	10.5
Mean length of lens fiber cell (mm)	4
Mean growth rate after birth (cells/year)	3 × 10^4^
Cell number in the lens epithelium	3 × 10^5^
Cell number in the posterior subcapsular region	3 × 10^5^
Cell number in the lens cortex	6 × 10^5^
Cell number in the lens nucleus at birth	6 × 10^5^
Stem cell fraction in the lens epithelium (%)	0.8
Stem cell number in the lens epithelium	2,400
Annual fraction of divided cells in the lens epithelium	0.1

The initial number of LECs in the lens epithelium at birth was set as 3 × 10^5^ cells (roughly corresponding to the number of LECs calculated based on the mean LEC density of 5652 cells/mm^2^ in the lens epithelium [22] and the size of the lens at 1–40 years of age [19]) (Table 1). Lacking any other information, the initial number of LFCs in the PSC region at birth was assumed to be the same as that of LECs in the lens epithelium, i.e., 3 × 10^5^ cells (Table 1). The number of LFCs in the lens cortex, embryonic, and fetal lens nuclei has been reported to be 6.65 × 10^5^, 8 × 10^2^, and 7 × 10^5^ cells, respectively [20]. Therefore, rounding these numbers, we set the initial number of LFCs in the lens cortex and in the lens nucleus at birth as 6 × 10^5^ cells each (Table 1), which are therefore simple multiples (20 ×) of the LFC growth rate of 3 × 10^4^ cells/year. The surface area of the human lens increases with age, but its extent is <70% from age 1 year to 90 years [19]). It was hence assumed that whereas the number of LECs in the lens epithelium and LFCs in the PSC region and the lens cortex remains unchanged from birth onwards, the number of LFCs in the lens nucleus begins with 6 × 10^5^ cells and then increases at 3 × 10^4^ cells/year.

We assume that (*i*) a stem cell asymmetrically divides into a stem cell and a progenitor cell (i.e., LEC), (*ii*) a progenitor cell (i.e., LEC) symmetrically divides into two progenitor cells, and (*iii*) stem cell divisions and progenitor cell (i.e., LEC) divisions occur at the same annual rate within the limited compartment [23]. The following equation therefore applies:

$$N_{T}(n)=N_{S}\times 2^{n},$$ (2)

where *N*_T_(*n*) is the total cell number in the lens epithelium after *n* cell divisions (e.g., 3 × 10^5^ cells at birth), *N*_S_ is the number of stem cells, and *n* is the number of stem cell divisions as well as that of progenitor cell divisions [24]. Under these conditions, when stem and progenitor cells divide 7 times, from Eq (2), the number of stem cells *N*_S_ = 3 × 10^5^/2^7^ = 2344 ≈ 2.4 × 10^3^ (rounded up). The implied stem cell fraction in the lens epithelium cell population at birth = (the stem cell number of 2.4 × 10^3^ cells)/(the total cell number in the lens epithelium of 3 × 10^5^ cells) = 0.8%, similar to the side population cell fraction of about 1% in the mouse lens [25]. In this scenario, the fraction of divided cells (λ) at birth becomes 0.1 [(the growth rate of 3 × 10^4^ cells/year)/(the total cell number in the lens epithelium of 3 × 10^5^ cells)].

The numerical values for above-mentioned various parameters should vary among individual human lenses, although the only significant determinant of the lens weight is age [26]. For instance, the mean cross-section of 10.5 μm^2^ is assumed for all LFCs here (Table 1), but cross-sections of LFCs at juvenile and adult lens nuclei were reported as 14 ± 5 and 7 ± 2 μm^2^, respectively [20]. The potential inter- and intra-individual differences were recognized, but for simplicity, the present simplified cell population model uses a single set of the numerical values for each parameter as listed in Table 1.

## Development of a model for spontaneous cataractogenesis

### Three fundamental principles considered to develop a model

To the best of our knowledge, no biomathematical models that can reproduce the human cataract data have been reported hitherto. Here, we attempted to develop a mathematical model with minimal parameters for the analysis of human cataract data. To develop such a model for cataractogenesis, three fundamental principles (basic assumptions) were considered.

**Fig 2:**
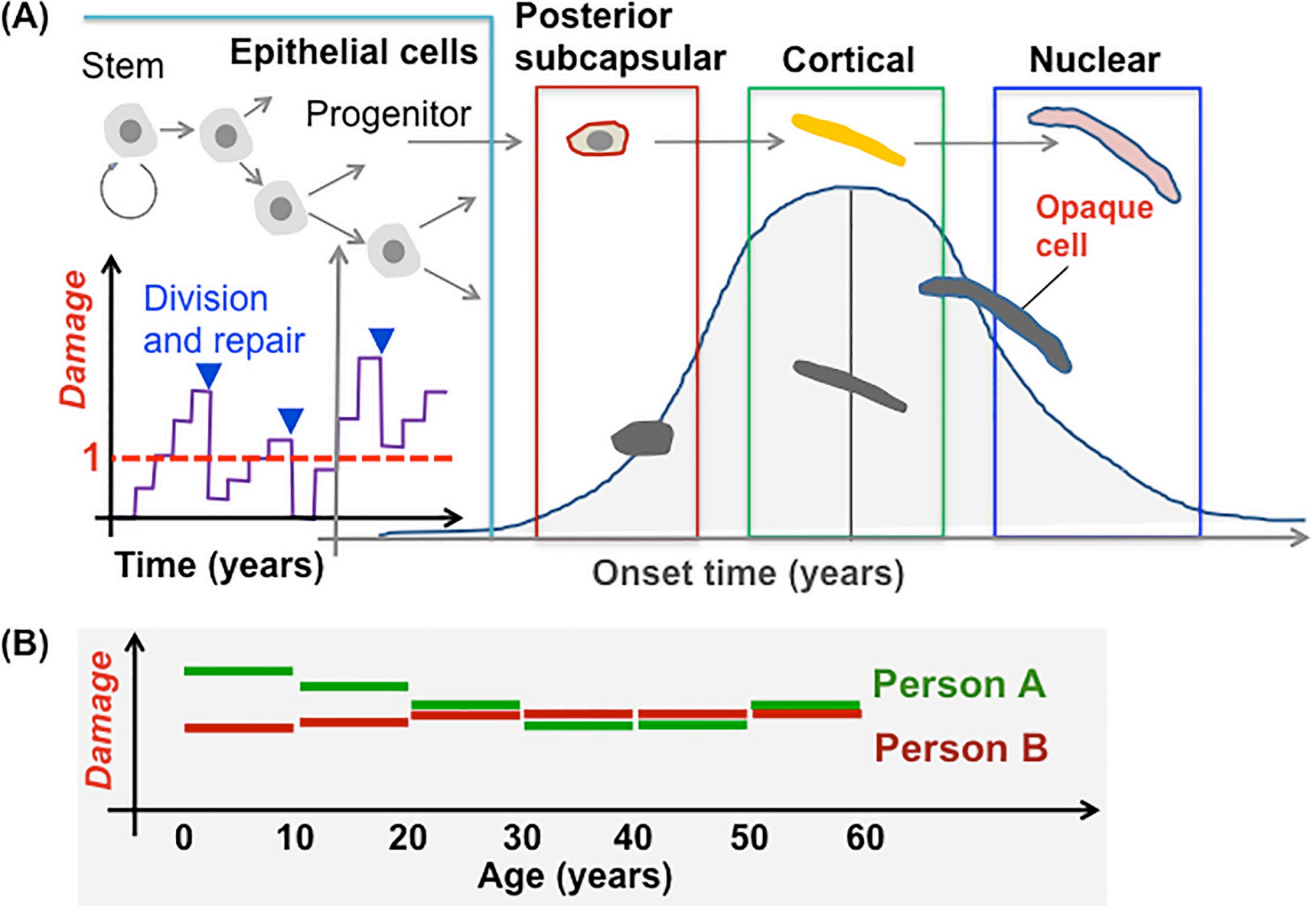
A schematic diagram outlining a model for spontaneous cataractogenesis. (A) Modeling of processes leading to manifestations of cataract predicated on the level of *Damage* that accumulates in lens epithelial cells. On one hand, upon cell division, the level of *Damage* decreases due to repair. On the other hand, when the level of *Damage* continues to exceed 1, posterior subcapsular or cortical cataracts appear at the onset time assigned to individual cells. (B) Lifelong assignment of *Damage*. *Damage* is randomly assigned to each individual person for every decade of life. The panel exemplifies two persons assigned with different levels of *Damage* as a function of age in years.

The first fundamental principle was that “*Damage*” to LECs in the lens epithelium leads to lenticular opacification. In support of this principle, the lens epithelium has been considered as the most plausible target tissue for IR cataractogenesis, among which LECs in the GZ have been regarded as the most relevant cells at risk, because cataracts occur in animal models after the equatorial region is locally irradiated, but not after irradiation when the GZ is shielded [27–30]. The term *Damage* is used here to broadly mean any cause of lenticular opacification, such as DNA damage, excessive proliferation, and abnormal differentiation [31]. Considering the first fundamental principle, a model assumed that (*i*) *Damage* accumulates only in progenitor cells in the lens epithelium, (*ii*) *Damage* is passed in equal measure to all daughter cells of each dividing progenitor cell, and (*iii*) cells become opaque when *Damage* continues to exceed a threshold *Damage*_Thresh_ and when the onset time comes (Fig 2A).

The second fundamental principle was that cells possess *Damage* repair machinery. A model thence assumed *Repair* that reduces *Damage* (Fig 2A). However, once *Damage* exceeds the threshold *Damage*_Thresh_, the repair machinery is irrelevant.

The third fundamental principle was that a personal history for *Damage* accumulation and cataract onset differs among individuals considering that human epidemiology data are population data. In our model, a personal *Damage* history was determined for each individual, where *Damage* was generated with random numbers that change every decade (Fig 2B).

Next, a model with a minimal set of parameters based on these three fundamental principles was used to reproduce the Beaver Dam Eye Study (BDES) data [32]. The threshold *Damage*_Thresh_ is a relative number, and was therefore set as 1. Then, the following five free parameters were considered: (*i*) the mean level of *Damage* to cells considering a personal *Damage* history that changes among individuals and with age in each individual (every decade), (*ii*) standard deviations (SDs) of such *Damage* level, (*iii*) the level of *Damage* repair, (*iv*) the mean time from when cell composing the lens exceeds a threshold until cataract onset, and (*v*) SDs of such onset time, which are referred hereafter to as *mDamage* (per year), *sdDamage* (per year), *Repair* (per progenitor cell division), *mOnset* (years), and *sdOnset* (years), respectively.

*Repair* was assumed for calculation purposes to occur once only per cell division cycle, during a progenitor cell division. Corresponding to *Damage*_Thresh_ = 1, we assumed that the maximal level of *Damage* repair, *Repair*, was 1, and indeed that *Repair* was identically equal to 1. Repair was subtracted from *Damage* upon progenitor cell division. For *Damage*, UV exposure represents one of the major causes of spontaneous cataractogenesis, but its personal dosimetry information is readily unavailable. In addition, the BDES data are available for every 10 years of age. Hence, *Damage* reflecting a personal *Damage* history was changed every 10 years of age with random numbers drawn from a normal distribution ~ max[*N*(*mDamage*, *sdDamage*^2^), 0]. The onset distribution (in years) was likewise assumed to be ~ max[*N*(*mOnset*, *sdOnset*^2^), 0], i.e., is truncated at 0. We assume that:

sdDamage=0.2×mDamage, (3)

sdOnset=0.2×mOnset. (4)

It should be noted that the max[] is needed here to formally ensure non-negativity of these two quantities, but in practice numbers drawn from these normal distributions will almost certainly (with probability >1–10^−23^) be positive.

For programing of a model based on the aforedescribed three fundamental principles, the platform of the NetLogo software was used, which is freely downloadable at http://ccl.northwestern.edu/netlogo/. NetLogo is a java platform that has been used to analyze complex systems, and adopts the multiagent modeling Logo languages [33]. All the source code files for NetLogo used in this study are provided in S1 Appendix. Cell population dynamics was simulated with a simplified cell population model, but at a scale of 1:100 (i.e., assuming 24 stem cells in the lens epithelium at age 0 year for each individual person). Calculations were made at one year intervals, with *Damage* taking the given (fixed) value for the specified decade (and *Repair* = 1 throughout). Cell migration from the lens epithelium to other lenticular compartments was evaluated once per year. A calculation for one person was completed once a PSC or cortical cataract was manifested, followed by a calculation for another person. A state of opacification was called a PSC cataract and a cortical cataract, respectively, when the opaque cell fraction among all cells each in the PSC region and in the lens cortex exceeds 5% (the same criteria as those used in the BDES [32]).

### Determination of the optimal values of the model parameters for reproducing the BDES data

The optimal values of *mDamage*, *mOnset* and *sdOnset* that can reproduce the BDES data [32] were determined based on the residual sum of squares (RSS) between the trial calculation results and the BDES data. This was done by fixing all random numbers at a given set and minimizing the RSS for the given particular (deterministic) implementation of the quasi-stochastic model. To this end, the following three step trial calculations were made.

**Fig 3:**
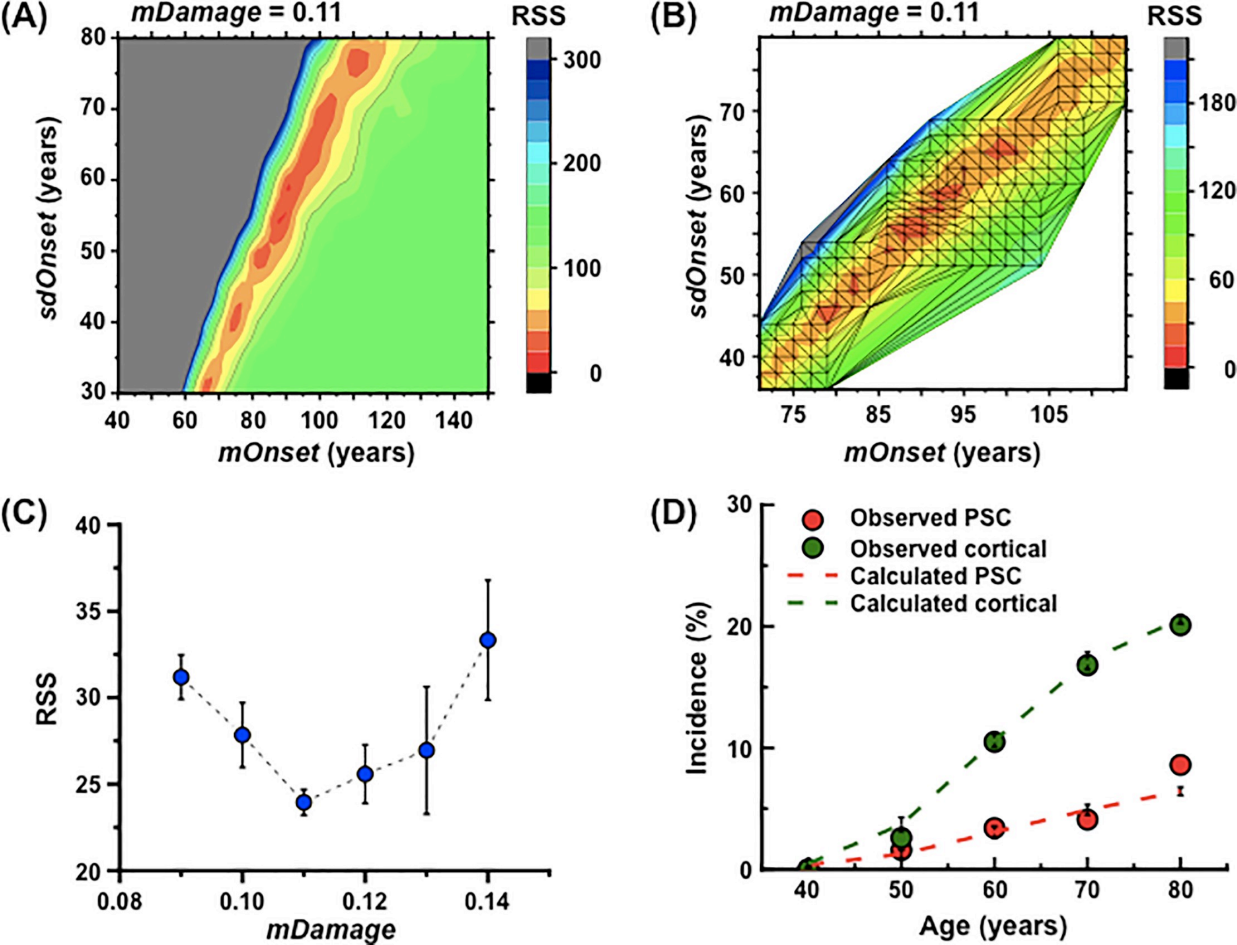
Determination of the optimal values of the model parameters for reproduction of the Beaver Dam Eye Study (BDES) data. (A) Changes in the residual sum of squares (RSS) from the BDES data [32] as a function of onset time. The means and standard deviations (SDs) of the onset time (*mOnset* and *sdOnset*, respectively) were calculated at five year intervals each for 40–150 years and 30–80 years in 300 lenses, where the level of mean *Damage* (*mDamage*) was set as 0.07–0.16 at 0.01 intervals (the data shown here for *mDamage* of 0.11). Then, the RSS vs the BDES data was calculated. (B) Changes in the RSS as a function of onset time. *mOnset* and *sdOnset* were calculated at two year intervals in 1500 lenses, where *mDamage* was set as 0.09–0.14 at 0.01 intervals (the data shown here for *mDamage* of 0.11). Then, the RSS vs the BDES data was calculated. These ranges of onset time were selected from the low RSS area in panel A. (C) Changes in the RSS as a function of *mDamage*. The grids were added onto the result in panel B, and the means and SDs of RSS in top 10 grids with low RSS were plotted at each *mDamage*. (D) Comparison of the cataract incidence between the calculated data and the observed BDES data. Simulations were conducted for 3000 lenses at *mOnset* of 89 years, *sdOnset* of 56 years, and *mDamage* of 0.11. Red and green circles show the BDES data for PSC and cortical cataracts, respectively. Red and green dotted lines show the calculated data for PSC and cortical cataracts, respectively. The calculated data are presented as means and 95% confidence intervals (CIs).

First of all, because the BDES data show that cataract incidence reaches 70% at age ≥75 years [32], the first trial calculation was made for 300 lenses at *mOnset* of 40–150 years and *sdOnset* of 30–80 years, both at five year intervals, and for each *mDamage* set at 0.01 intervals in a range between 0.07 and 0.16. Within the low RRS area, there was the very low RRS area suggestive of the optimal parameter values (Fig 3A and S1 Fig). The grids were added onto the results, revealing that less than two grids have RSS ≤30 at *mDamage* of ≤0.08 and ≥0.15. Therefore, *mDamage* was narrowed down to 0.09–0.14.

The second trial calculation was made for 1500 lenses at *mOnset* and *sdOnset* in the low RSS area observed in the first trial calculation, both at two year intervals, and for each *mDamage* set at 0.01 intervals in a range between 0.09 and 0.14 (Fig 3B and S2 Fig). The grids were added onto the results, and the means and SDs of RSS in top 10 grids with low RSS were plotted at each *mDamage* (Fig 3C). The mean RRS was found to become lowest at *mDamage* of 0.11 (Fig 3C).

The third trial calculation was made for 3000 lenses at *mOnset* of 85–95 years and *sdOnset* of 53–62 years, both at one year intervals, and at *mDamage* of 0.11, revealing that RSS becomes minimal (RSS = 17.25) at *mOnset* of 89 years and *sdOnset* of 56 years (S3 Fig).

Fig 3D shows the comparison of incidence of PSC and cortical cataracts as a function of age, between the BDES data and the data calculated with aforementioned optimal parameter values (i.e., *mOnset* of 89 years, *sdOnset* of 56 years, and *mDamage* of 0.11). These results demonstrate that our model can reproduce the BDES data. Intriguingly, the analysis of a personal history revealed that whilst *Damage* for PSC cataracts peaks at or within a decade prior to onset (S4 Fig), *Damage* for cortical cataracts peaks at two decades prior to onset (S5 Fig).

Because our model is based on Monte Carlo simulations, the results are intrinsically stochastic. The Monte Carlo errors of each simulation are expressed as 95% confidence intervals (CIs), unless otherwise specified. It should be noted that these errors do not take account of the uncertainties involved in estimating model parameters.

### The sensitivity of *sdDamage* and *Repair* to the incidence of PSC and cortical cataracts

**Fig 4:**
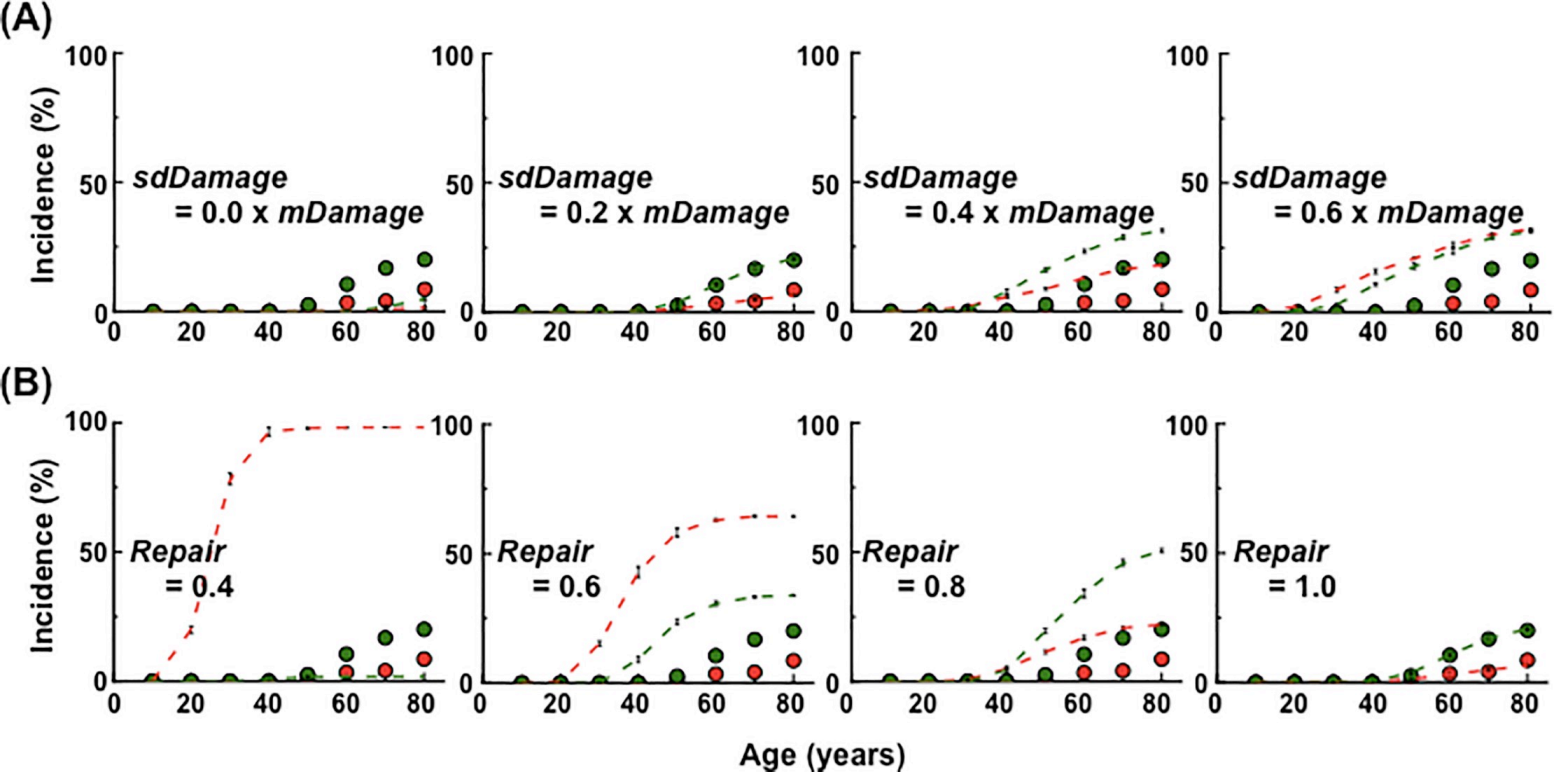
Changes in the simulation results by parameter values. (A) Comparison of the cataract incidence between the calculated data and the Beaver Dam Eye Study (BDES) data [32], when the standard deviations (SDs) of *Damage* (*sdDamage*) are set as 0.0, 0.2, 0.4 or 0.6 each multiplied by *mDamage*. (B) Comparison of the cataract incidence between the calculated data and the BDES data, when *Repair* is changed from 0.4 to 1.0 at 0.2 intervals. Red and green circles show the BDES data for posterior subcapsular (PSC) and cortical cataracts, respectively. Red and green dotted lines show the calculated data for PSC and cortical cataracts, respectively. The calculated data represent means and 95% confidence intervals (CIs). For parameter determinations made in Fig 3 and S1–S3 Figs, *sdDamage* of 0.2 × *mDamage* and *Repair* of 1 were used.

Fig 3A–3C and S1–S3 Figs show that a departure from the BDES data occurs depending on the levels of changes in the numerical values of *mOnset* and *sdOnset*. We hence examined the impact of changes in parameter values of *sdDamage* and *Repair*. On one hand, the incidence of both PSC and cortical cataracts increased with increasing *sdDamage* (Fig 4A); c.f., for determination of the optimal values, 0.2 of *mDamage* was set as *sdDamage* to introduce individual differences in the level of *Damage*. On the other hand, the incidence of PSC cataracts decreases and its onset occurs at younger age with decreasing *Repair*; in contrast, however, the incidence of cortical cataracts is higher at *Repair* of 0.8 and 0.6 but lower at 0.4 than that at 1 (Fig 4B). These results show that while *sdDamage* affects the incidence of both PSC and cortical cataracts, *Repair* is a great contributor to the incidence of PSC cataracts rather than that to cortical cataracts.

## Application of a model for spontaneous cataractogenesis to ionizing radiation cataractogenesis

Our spontaneous cataractogenesis model is characterized by a combination of *Damage* accumulativeness, a *Damage* threshold and onset time, for which a set of the optimal parameter values were determined to reproduce the BDES data (Fig 3). We now test the applicability of this model to the human IR cataract data. For this purpose, *Damage* in an arbitrary unit needs to be converted to *Damage* in Gy (the unit of absorbed dose).

In 1957, Merriam and Focht reported cataracts in 97 radiotherapy patients with a follow-up period of ≤31 years [34]. The Merriam and Focht data [34] included information on dose, duration of treatment, time cataract first seen after the completion of radiotherapy (here called onset time), progressive or stationary nature of opacities, and the length of a follow-up period, for each of 97 patients, and therefore had long served as the most influential scientific basis for the ICRP equivalent dose limit for the lens of 150 mSv/year [12]. Merriam and Focht [34] divided these 97 cases into three groups depending on the duration of treatment: 20 were defined as single treatments (called group 1), 49 as treatments from 3 weeks to 3 months (group 2), and 28 as treatments from 3 months to 9 years (group 3). Of these, the data used here were for 41 patients at age ≥30 years at the time of exposure in groups 1 and 2. In contrast to more standard statistical evaluations of risk (e.g., by fitting a Cox proportional hazards model [35]), there is no mechanistic information available as to how IR cataractogenesis adds to spontaneous cataractogenesis [7]. Nevertheless, we assumed that:

$$Damage={Damage}_{Spontaneous}+{Damage}_{Radiation},$$ (5)

i.e., that the total was the sum of spontaneous *Damage* (*Damage*_Spontaneous_) and IR-associated terms (*Damage*_Radiation_), all in arbitrary units. *Damage*_Radiation_ was assumed to be proportional to absorbed dose *D* (Gy), so that:

$${Damage}_{Radiation}=R\times D$$ (6)

for some constant *R*. In our model, the *Damage*_Radiation_ is evaluated (with the spontaneous term *Damage*_Spontaneous_) once a year, independent of the dose rate.

**Fig 5:**
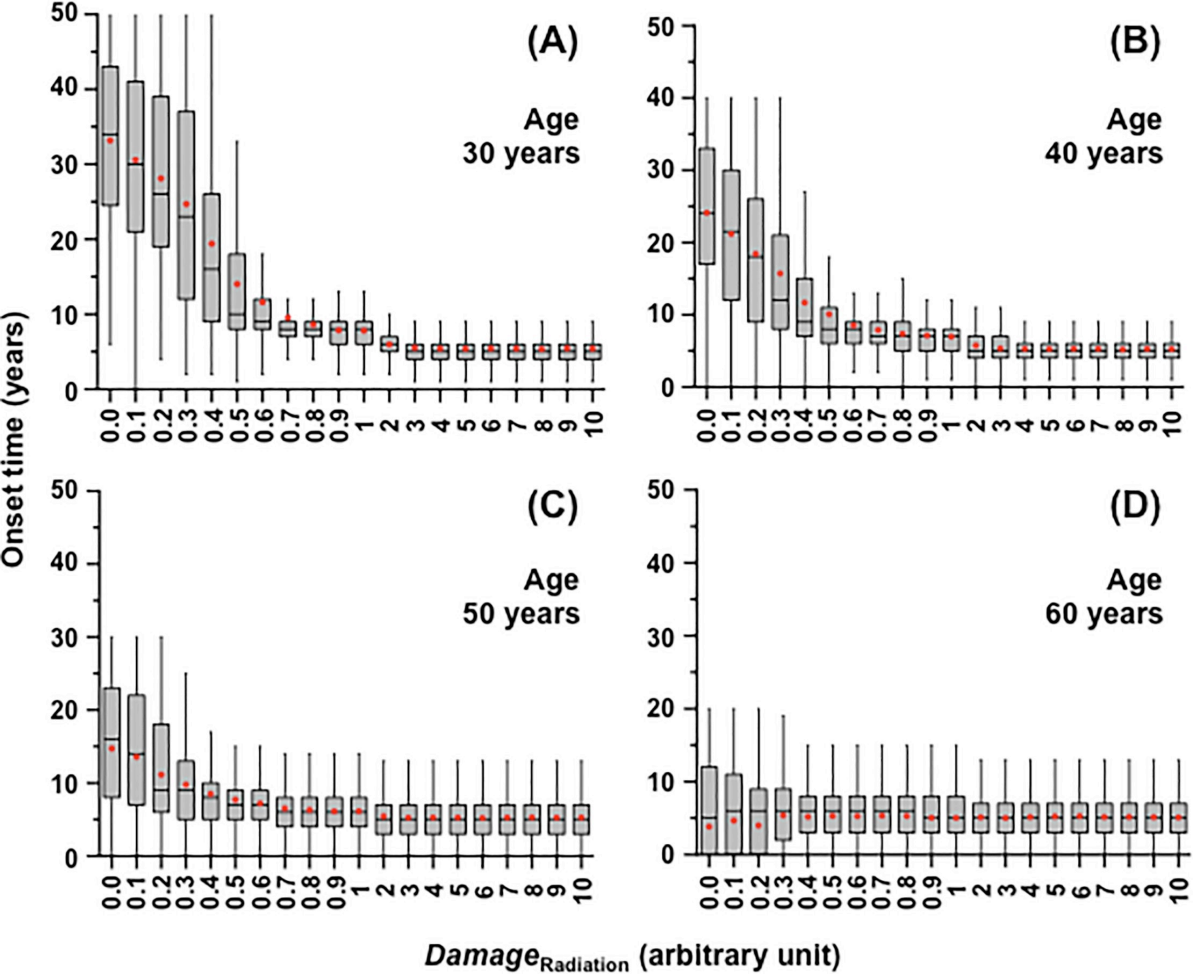
The simulation of cataract onset time after ionizing radiation *Damage* (*Damage*_Radiation_). The simulated onset time for posterior subcapsular (PSC) cataracts following exposure at age 30, 40, 50 and 60 years as a function of *Damage*_Radiation_ (in an arbitrary unit) is shown in box-and-whisker plots in panels (A), (B), (C) and (D), respectively. The box indicates the inter-quartile range containing middle 50% of the data. The horizontal line within the box, and the vertical line (whisker) beyond the box hinges indicate the median value, and the data within 1.5 times the inter-quartile range, respectively. Red dots indicate the means.

The time in years from exposure to *Damage*_Radiation_ (0–10 in an arbitrary unit) at 30, 40, 50 or 60 years of age until onset of a PSC cataract (i.e., onset time) was calculated for 1000 lenses (Fig 5). Onset time decreased with increasing *Damage*_Radiation_, but then became nearly constant from about 0.5 onwards. This was because our model assumes that only cells in the lens epithelium receive and accumulate *Damage*. A certain period of time is therefore necessary from when cells in which a threshold is exceeded are significantly increased in the lens epithelium, until when opaque cells exceed 5% in the PSC region. Our model estimates such a latency period of 5 years (derived from median onset time at *Damage*_Radiation_ of ≥1 in Fig 5A and 5B). Taken together, the extent of a decrease in onset time becomes smaller as age at exposure increases, because the incidence of spontaneous PSC cataracts increases at age >50 years, and thence becomes indistinguishable from IR PSC cataracts.

**Fig 6:**
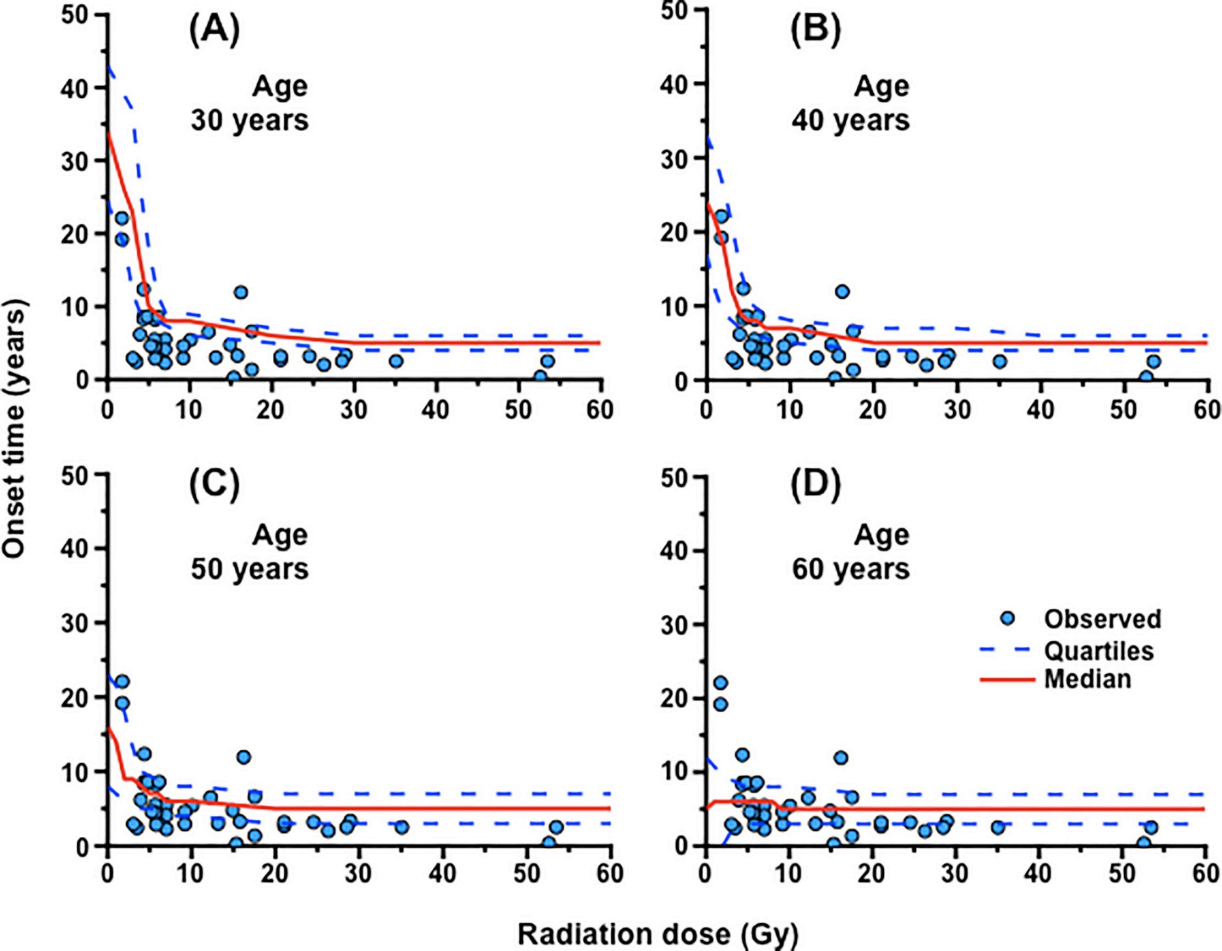
Comparison of the cataract onset time between the Merriam and Focht data [34] and the simulated data. The simulated onset time for posterior subcapsular (PSC) cataracts following exposure at age 30, 40, 50 and 60 years as a function of dose assuming ionizing radiation *Damage* (*Damage*_Radiation_) of 0.1/Gy is shown in panels (A), (B), (C) and (D), respectively. The Merriam and Focht data shown as blue circles are for all corresponding data in those at age ≥30 years at the time of exposure in groups 1 and 2. For the calculated data, red solid lines indicate the median values, and blue dotted lines indicate the first and third quartiles (i.e., the 25th and 75th percentile).

The Merriam and Focht data [34] also exhibited the similar shape of the dose response for onset time, i.e., a dose dependent decrease followed by little change (Fig 6), as observed in Fig 5. On one hand, onset time was calculated for each age at exposure at various levels of *Damage*_Radiation_. On the other hand, the Merriam and Focht data [34] were limited in terms of the number of cases and age distribution, which rendered it impossible to make a plot precisely for each age. Instead, we set out to determine the conversion coefficient *R* from *Damage*_Radiation_ (in an arbitrary unit) to dose (Gy) by following three steps. First, the calculated data (left panels in S6 Fig) were fitted to the following equation:

$$T=T_{Min}+T_{Dose}e^{–\frac{D}{D_{0}}},$$ (7)

where *T*, *T*_Min_, *T*_Dose_, *D* and *D*_0_ are onset time (years), minimum onset time (years), dose-dependent onset time (years), *Damage*_Radiation_ (in an arbitrary unit) and slope, respectively. Background onset time at *D* = 0 is *T*_Min_ + *T*_Dose_. Second, the Merriam and Focht data (right panels in S6 Fig) were fitted to the same equation, but with the *T*_Dose_ value obtained from the calculated data and *D* indicating dose (Gy). Third, *R* was calculated as *D*_0_ for the Merriam and Focht data (*D*_0 Obs_) divided by *D*_0_ for the calculated data (*D*_0 Cal_). The *R* value of 9.300 ± 1.803 (yielded from the *R* values at age 40, 50 and 60 years shown in S6 Fig) was rounded to determine *R* as 10 (Gy/*Damage*_Radiation_). Thus, 1 *Damage*_Radiation_ was converted to 10 Gy for comparison between the Merriam and Focht data and the calculated data. With this approach, the dose response for onset time was obtained (Fig 6).

Finally, with a set of parameter values determined to reproduce the BDES data and the Merriam and Focht data, our model was applied to estimate the incidence of PSC and cortical cataracts following chronic lifetime exposure where exposures occur at a constant dose rate from birth to age 80 years. There was little if any change in cataract incidence at a lifetime cumulative dose of 0, 0.1 (1.25 mGy/year) and 1 Gy (12.5 mGy/year) (Fig 6A–6C). However, cataract incidence was elevated at age ≥40 years at a lifetime cumulative dose of 10 Gy (125 mGy/year) (Fig 6D), and such an increase in cataract incidence at age 80 years was observed at a lifetime cumulative dose of ≥2 Gy (25 mGy/year) (S7 Fig).

## Discussion

**Fig 7:**
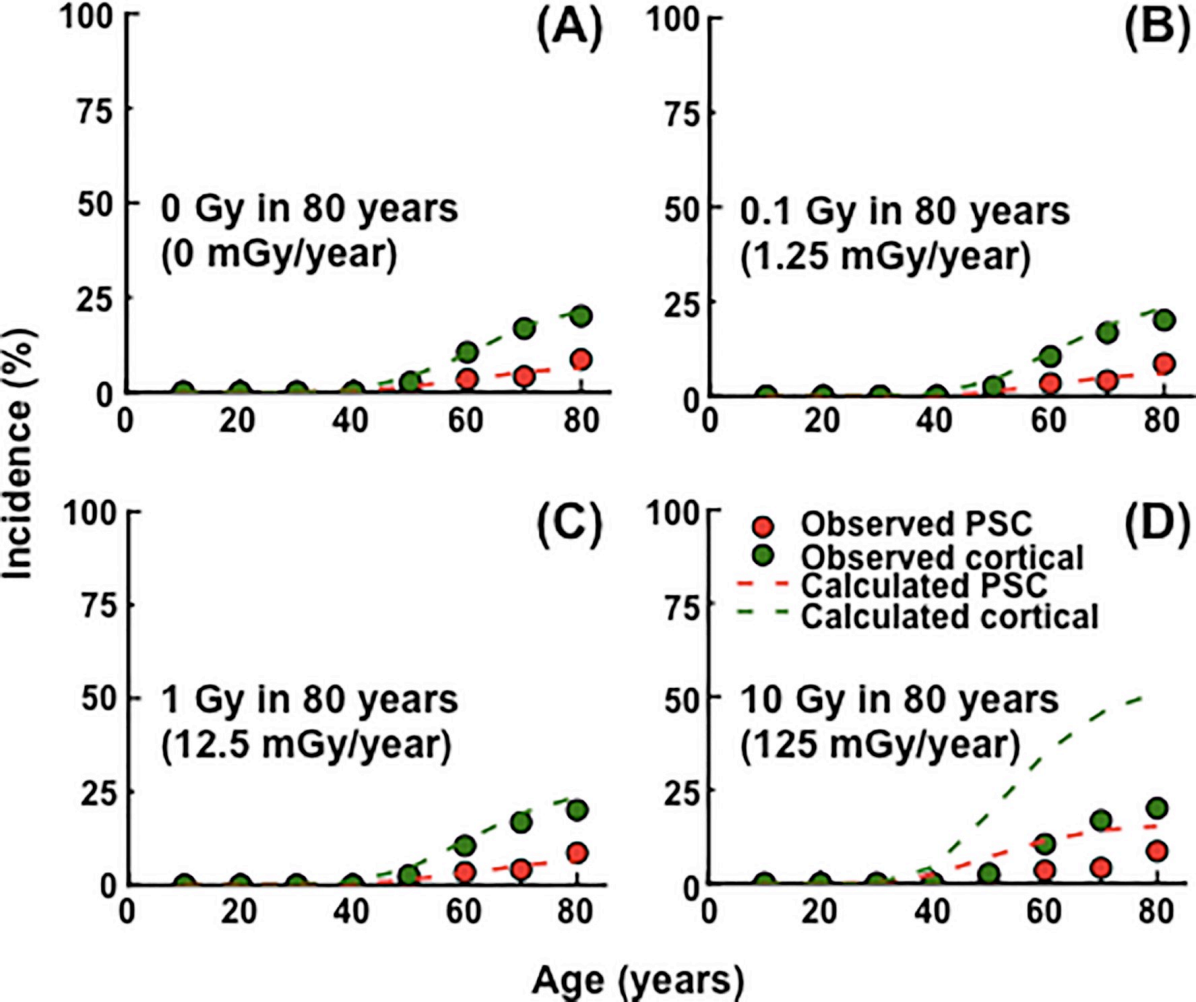
Changes in the cataract incidence following lifetime chronic exposure. Exposures were assumed to occur at age 0–80 years at a constant dose rate that gives a lifetime cumulative dose of 0, 0.1, 1 and 10 Gy at age 80 years. Cataract incidence following such lifetime chronic exposure to 0, 0.1, 1 and 10 Gy is shown in panels (A), (B), (C) and (D), respectively. Red and green circles show the BDES data for posterior subcapsular (PSC) and cortical cataracts, respectively. Red and green dotted lines show the calculated data for PSC and cortical cataracts, respectively.

We have here developed a simplified cell proliferation model for human lens growth predicated on proliferation of stem and progenitor cells as well as differentiation of LECs into LFCs (Fig 1 and Table 1), followed by development of an *in silico* model for cataractogenesis. Our cataractogenesis model assumed that *Damage* to LECs in the lens epithelium causes cataracts, cells possess repair that reduces *Damage*, and a personal *Damage* history differs among individuals (Fig 2). Five free parameters (i.e., *mDamage*, *sdDamage*, *Repair*, *mOnset* and *sdOnset*) were employed, although there are a number of other variables in the model, not fitted to data, for example those controlling the initial stem cell population and rates of transfer between compartments. *Damage* was inter-individually changed every decade, and a *Damage* threshold (also maximal *Repair*) was set as 1 (Fig 2). A model for spontaneous cataractogenesis with parameter values optimized to reproduce the BDES data (Figs 3 and 4 and S1–S5 Figs) was applied to IR cataractogensis for reproducing the Merriam and Focht data (Figs 5 and 6 and S6 Fig) and for estimating cataract incidence following chronic lifetime exposure (Fig 7 and S7 Fig).

Cataract is thought to result from cumulative oxidative stress in the eye lens [36], one component of which is associated with cumulative UVA exposure, as UVA is thought more directly capable of penetrating to the lens than UVB [37]. As such, one may identify the *Damage* variable as some measure of cumulative oxidative stress, and the *Repair* variable some of the known cellular mechanisms for countering oxidative damage [38,39]. The term *Damage* was used in the present study to mean any cause of cataractogenesis such as DNA damage, excessive proliferation, abnormal differentiation, denaturation of lens protein and structural alterations in lens lipids, in addition to oxidative damage [6,7,13,40]. The degree of actual contribution of each mechanism to cataractogenesis, interactions among various mechanism, the dose response relationship for each mechanism, the temporal kinetics of damage production and repair, and damage accumulativeness remain unknown. Such identification clearly needs more extensive biological studies to give insights into (and validate) the biological basis for various assumptions in this study (the first and second fundamental principles in particular), but is not necessary for the purpose of our present simulation.

Various biological mechanisms can be considered in the model, which would necessarily lead to an increase in the number of parameters, with consequent danger of overfitting. The model developed here is very simple with a minimal set of parameters, which nevertheless seem capable of reproducing the BDES data [32] (Fig 3) and the Merriam and Focht data [34] (Fig 6). As such, the model may be adequately and parsimoniously parameterized. Undoubtedly, there are other cataract data to which the model could be fitted, e.g., in atomic bomb survivors [41], or the Chernobyl clean-up workers [42], but the dataset is unavailable in the form that would be needed to fit the present model: this is because the individual data for these cohort members have not been made publically available. Nonetheless, one could envisage possible further developments of the model, for example to model the effects of corticosteroids, diabetes, obesity and cigarette smoking, all known, or suspected, risk factors for cataract [3]. One might envisage extending the additive model for the *Damage* term, with extra additive terms for these risk factors, as given by Eqs (5) and (6).

We assumed a decade in which an individually varied *Damage* history was applied (Fig 2), and otherwise assumed a time scale of a year, during which lens growth, cell migration from the lens epithelium to other lenticular compartments, and *Damage* and *Repair* were all assumed to occur. Although there is little evidence that radiation dose rate appreciably affects cataract risk [43], conceivably the *Damage*_Radiation_ term might be modified to take account of the radiation dose rate within the year. Shorter timescales could be considered, with suitable scaling of all damage, repair and migration processes, although this would somewhat add to the computational burden. Such improvements in temporal resolution could make the model more applicable to various exposure situations, and could be used to explore dose fractionation effects. Related to this, we considered an IR exposure scenario in Fig 7 and S7 Fig of chronic lifetime exposure over the ages of 0–80 years, relevant to chronic environmental exposures, such as those in the residents of high natural background radiation areas (e.g., Kerala, India, and Yangjiang, China). A period of 50 years and 70 years is broadly used for occupational and public exposures from radiation protection viewpoints [44], and application of the model in such a timeframe will be useful to estimate cataract incidence following occupational or public exposures.

Our model did not consider cellular or tissue responses to IR and their dependence on dose and dose rate. In this regard, the radiosensitivity may differ not only between stem cells and progenitor cells, but also among the regions in the lens epithelium [45]. There may also be stem cell competition, e.g., between irradiated and non-irradiated stem cells [46]. IR should inactivate cells dose dependently, and our *in vitro* studies have shown that human LECs contain several subsets each with different IR responses: IR facilitates formation of larger clonogenic colonies in one subset via enhanced cell proliferation, but may also lead to formation of smaller abortive colonies in other subsets via induction of premature senescence [47,48]. Such enhanced proliferation we first observed in HLEC1 at ≥2 Gy *in vitro* [47] was then observed in a different human LEC cell line *in vitro* [49] and *in vivo* [45]. We have previously hypothesized that whereas these responses of LECs underlie IR PSC cataracts, acceleration of age-related changes such as denaturation of lens proteins underlie IR cortical cataracts [6]. IR-induced changes in lens proteins have been shown to occur at very high dose (e.g., several tens to thousands of Gy [50,51]), and have recently been shown to occur at 5 Gy *in vivo* [52]. ICRP judges that the primary target for IR PSC cataracts is LECs in the GZ of the lens epithelium [11], and our model assumed that only LECs in the lens epithelium receive and accumulate *Damage*. Though the target for IR cortical cataracts remains unidentified, assumption of *Damage* (e.g., denaturation of lens protein and structural alterations in lens lipids [40]) to LFCs may be plausible. A greater consideration of dose responses of such biological mechanisms could lead to improvement of the model.

We employed the BDES data on spontaneous cataracts [32], which had data on 2711 cataracts in a cohort of 3684 persons. As such, it is a relatively small dataset, and there are difficulties of interpretation because of the method of selection of the population taken for the two eye examinations that each subject had. However, there is information on some of the standard risk factors for cataract in this cohort, in particular smoking and solar UV exposure [53,54], although no use was made of this information here. The Merriam and Focht data on IR cataracts [34] were also employed here, because the data included information on dose, onset time, etc for each person. The data have served as one of the scientific bases for the ICRP equivalent dose limit for the lens of 150 mSv/year. Associations of IR exposures with PSC and cortical cataracts have thus far been repeatedly reported [11,43,55–57], with a single paper reporting that with nuclear cataracts [58]. Of these, however, the Merriam and Focht data [34] dealt only with PSC cataracts, and are even smaller than the BDES data, with information on only 97 patients. Hence, improvement of the model will need the use of additional epidemiological datasets on IR cataracts from various cohorts. It would be useful in particular to consider epidemiological datasets with a wide range of dose and, if possible, dose rates, a long follow-up period, and information on onset and progression of cataracts for various cataract types in each cohort member. Such candidate cohorts may include atomic bomb survivors for acute exposure [59,60] and Russian Mayak workers for chronic exposure [58,61,62]. More generally, the use of larger population-based datasets, having information on specific standard risk factors other than radiation will be needed to improve the model.

## Conclusions

This study is the first to report an *in silico* model for cataractogenesis. First, a simplified cell proliferation model was developed for human lens growth according to proliferation of stem and progenitor cells as well as differentiation of LECs into LFCs. Then, a model for spontaneous cataractogenesis was developed with minimal parameters of damage, repair and onset that were optimized to reproduce the BDES data on a relationship between age and cataract incidence. Finally, a model for IR cataractogenesis was developed that can reproduce the Merriam and Focht data on a relationship between dose and cataract onset at various ages, which was further applied to estimate cataract incidence following chronic lifetime exposure. The model established here is very simple with minimal parameters, which can serve as the foundation for further development of the risk-predictive model with additional considerations of various biological mechanisms and epidemiological datasets with a wide range of dose and dose rate. Such model will be useful to predict the risk not only from the context of occupational and public radiation protection, but also from the context of normal tissue complications following radiotherapy.

## Supporting information

S1 Appendix Zip archive containing the source code files for NetLogo (*.nlogo). The NetLogo code “Code1” was used for Fig 3A and S1 Fig, “Code2” for Fig 3B, 3C and 3D, S2 and S3 Figs, “Code3” for Fig 4A, “Code4” for Fig 4B, “Code5” for Figs 5 and 6 and S6 Fig, “Code6” for Fig 7 and S6 Fig, and “Code7” for S4 and S5 Figs. A table listing the name of parameters that appear in the text and its corresponding name used in the NetLogo code is also enclosed.
(ZIP)

S1 Fig Changes in the residual sum of squares (RSS) from the Beaver Dam Eye Study (BDES) data [32] as a function of onset time. The means and standard deviations (SDs) of the onset time (*mOnset* and *sdOnset*) were calculated at five year intervals each for 40–150 years and 30–80 years of age in 300 lenses, where the level of mean *Damage* (*mDamage*) was set as 0.07–0.16 at 0.01 intervals (the data shown here for *mDamage* of 0.07, 0.09, 0.11, 0.13 and 0.15). Then, the RSS vs the BDES data was calculated. The panel for *mDamage* of 0.11 shown here is the same one as shown in Fig 3A.
(TIFF)

S2 Fig Changes in the residual sum of squares (RSS) from the Beaver Dam Eye Study (BDES) data [32] as a function of onset time. The means and standard deviations (SDs) of the onset time (*mOnset* and *sdOnset*) were calculated at two year intervals in 1500 lenses, where the level of mean *Damage* (*mDamage*) was set as 0.09–0.14 at 0.01 intervals (the data shown here for *mDamage* of 0.09, 0.10, 0.11, 0.12 and 0.13). Then, the RSS vs the BDES data was calculated. The panel for *mDamage* of 0.11 shown here is the same one as shown in Fig 3B.
(TIFF)

S3 Fig Changes in the residual sum of squares (RSS) from the Beaver Dam Eye Study (BDES) data [32] as a function of mean *Damage* (*mDamage*). The calculation was made for 3000 lenses at *mOnset* of 85–95 years and *sdOnset* of 53–62 years, both at one year intervals, and at *mDamage* of 0.11.
(TIFF)

S4 Fig Personal history of *Damage* averaged over all cells for posterior subcapsular (PSC) cataracts. A personal history of *Damage* for PSC cataracts manifested at indicated age in years, among 3000 lenses calculated for Fig 3D. Red and open circles indicate PSC cataracts and non-cataracts, respectively. The data are presented as means and 95% confidence intervals (CIs). The opaque cell fraction among all cells in the PSC region is defined in this study to exceed 5% in PSC cataract cases, but below 5% in non-cataract cases.
(TIFF)

S5 Fig Personal history of *Damage* averaged over all cells for cortical cataracts. A personal history of *Damage* for cortical cataracts manifested at indicated age in years, among 3000 lenses calculated for Fig 3D. Red and open circles indicate cortical cataracts and non-cataracts, respectively. The data represent means and 95% confidence intervals (CIs). The opaque cell fraction among all cells in the lens cortex is defined in this study to exceed 5% in cortical cataract cases, but below 5% in non-cataract cases.
(TIFF)

S6 Fig Determination of conversion coefficient *R*. First, the calculated data in left panels were fitted to the following equation: *T* = *T*_Min_ + *T*_Dose_ e^–*D*/*D*o^, where *T*, *T*_Min_, *T*_Dose_, *D* and *D*_0_ are onset time (years), minimum onset time (years), dose-dependent onset time (years), *Damage*_Radiation_ (in an arbitrary unit) and slope, respectively. Second, the Merriam and Focht data (right panels) were fitted to the same equation, but with the *T*_Dose_ value obtained from the calculated data and *D* indicating dose in Gy. Last, *R* was calculated as *D*_0_ for the Merriam and Focht data (*D*_0 Obs_) divided by *D*_0_ for the calculated data (*D*_0 Cal_), each for age 30, 40 and 50 years (panels A–C). The means and standard errors (SEs) of the yielded values for *T*_Dose_, *D*_0 Cal_, *D*_0 Obs_ and *R* are shown in each panel. For error bars in S6A–S6C Fig, see those in Fig 6A–6C.
(TIFF)

S7 Fig Changes in the cataract incidence at age 80 years following lifetime chronic exposure. Exposures were assumed to occur at age 0–80 years at a constant dose rate of 1.25, 12.5, 25, 37.5, 50, 62.5, 87.5 and 125 mGy/year, respectively that gives a lifetime cumulative dose of 0.1, 1, 2, 3, 4, 5, 7 and 10 Gy at age 80 years. Red and green circles show the calculated data for posterior subcapsular (PSC) and cortical cataracts, respectively.
(TIFF)
