# Supplementary material for: A biologically based mathematical model for spontaneous and ionizing radiation cataractogenesis
Source: PLoS One. 2019 Aug 23;14(8):e0221579. doi: 10.1371/journal.pone.0221579 (PMC6707595; doi:10.1371/journal.pone.0221579)
Supplement: S1 Appendix — The NetLogo code “Code1” was used for Fig 3A and S1 Fig, “Code2” for Fig 3B, 3C and 3D, S2 and S3 Figs, “Code3” for Fig 4A, “Code4” for Fig 4B, “Code5” for Figs 5 and 6 and S6 Fig, “Code6” for Fig 7 and S6 Fig, and “Code7” for S4 and S5 Figs. A table listing the name of parameters that appear in the text and its corresponding name used in the NetLogo code is also enclosed. (ZIP) [file pone.0221579.s001.zip › S1_Appendix/ParameterNameList.pdf]

The name of parameters in the text and its corresponding name in the NetLogo code.

The name in the text	The name in the code
mDamage	para_mean_dam
sdDamage	para_sd_dam
mOnset	mean_time_of_onset
sdOnset	sd_time_of_onset
Repair	repair_hr
age	age
Damage ($Damage_{\text{Spontaneous}}$)	damage
$Damage_{\text{Radiation}}$ ($R \times D$)	rad_dam
Lenses (total number of lenses)	n_person
