# Supplementary figures and images for: A biologically based mathematical model for spontaneous and ionizing radiation cataractogenesis
Source: PLoS One. 2019 Aug 23;14(8):e0221579. doi: 10.1371/journal.pone.0221579 (PMC6707595; doi:10.1371/journal.pone.0221579)

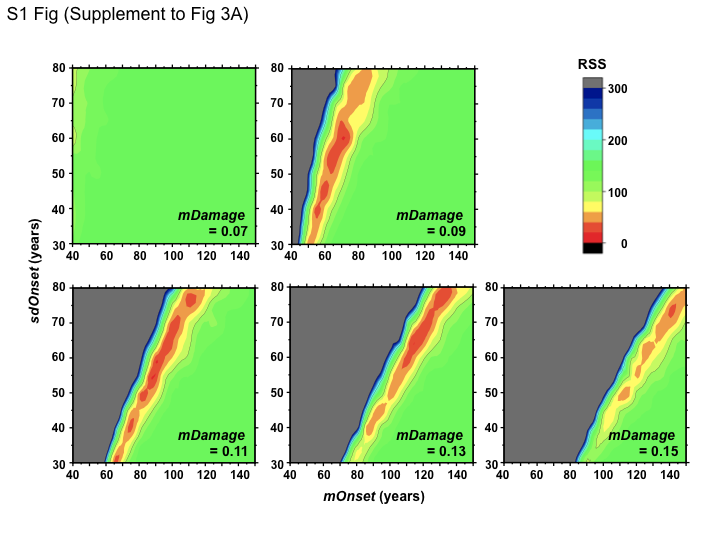

Supplement: S1 Fig — The means and standard deviations (SDs) of the onset time (mOnset and sdOnset) were calculated at five year intervals each for 40–150 years and 30–80 years of age in 300 lenses, where the level of mean Damage (mDamage) was set as 0.07–0.16 at 0.01 intervals (the data shown here for mDamage of 0.07, 0.09, 0.11, 0.13 and 0.15). Then, the RSS vs the BDES data was calculated. The panel for mDamage of 0.11 shown here is the same one as shown in Fig 3A. (TIFF) [file pone.0221579.s002.tiff]

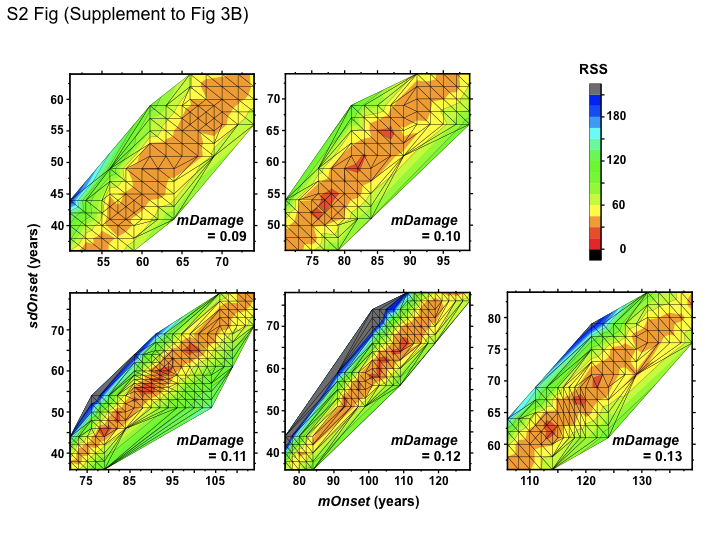

Supplement: S2 Fig — The means and standard deviations (SDs) of the onset time (mOnset and sdOnset) were calculated at two year intervals in 1500 lenses, where the level of mean Damage (mDamage) was set as 0.09–0.14 at 0.01 intervals (the data shown here for mDamage of 0.09, 0.10, 0.11, 0.12 and 0.13). Then, the RSS vs the BDES data was calculated. The panel for mDamage of 0.11 shown here is the same one as shown in Fig 3B. (TIFF) [file pone.0221579.s003.tiff]

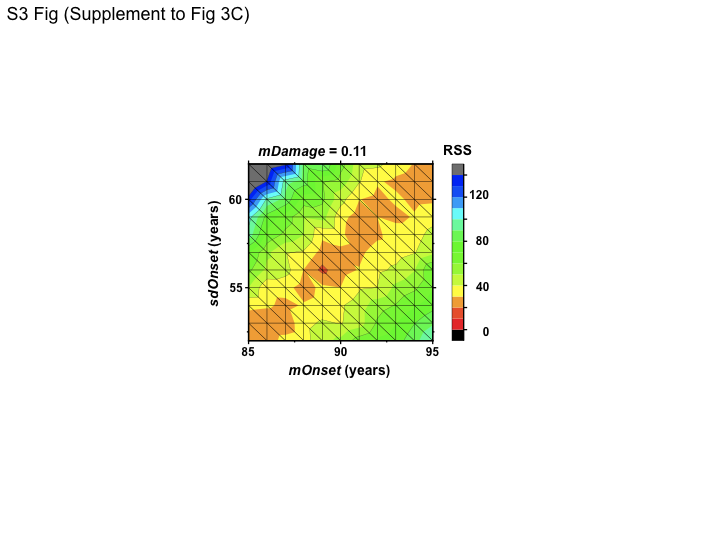

Supplement: S3 Fig — The calculation was made for 3000 lenses at mOnset of 85–95 years and sdOnset of 53–62 years, both at one year intervals, and at mDamage of 0.11. (TIFF) [file pone.0221579.s004.tiff]

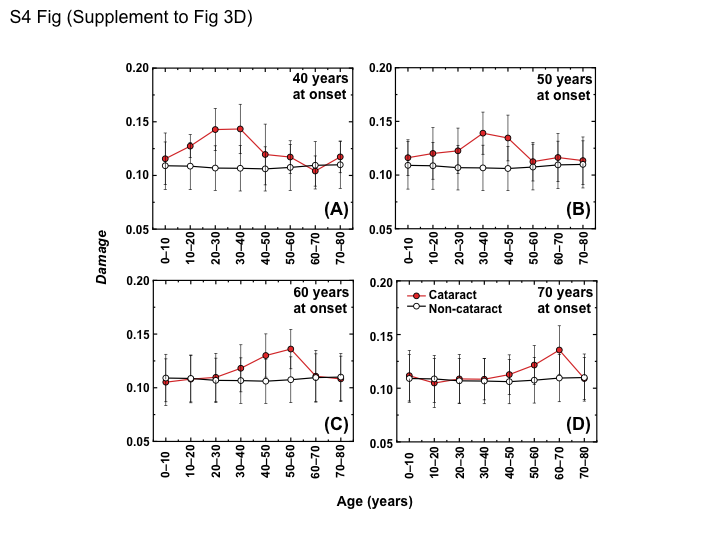

Supplement: S4 Fig — A personal history of Damage for PSC cataracts manifested at indicated age in years, among 3000 lenses calculated for Fig 3D. Red and open circles indicate PSC cataracts and non-cataracts, respectively. The data are presented as means and 95% confidence intervals (CIs). The opaque cell fraction among all cells in the PSC region is defined in this study to exceed 5% in PSC cataract cases, but below 5% in non-cataract cases. (TIFF) [file pone.0221579.s005.tiff]

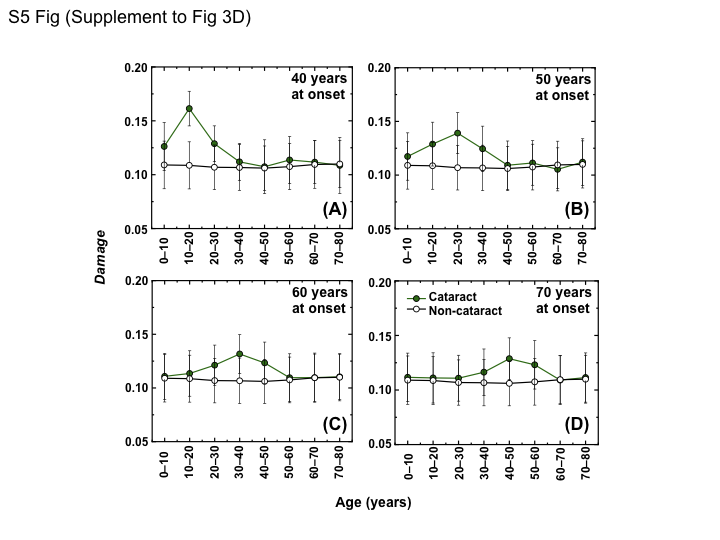

Supplement: S5 Fig — A personal history of Damage for cortical cataracts manifested at indicated age in years, among 3000 lenses calculated for Fig 3D. Red and open circles indicate cortical cataracts and non-cataracts, respectively. The data represent means and 95% confidence intervals (CIs). The opaque cell fraction among all cells in the lens cortex is defined in this study to exceed 5% in cortical cataract cases, but below 5% in non-cataract cases. (TIFF) [file pone.0221579.s006.tiff]

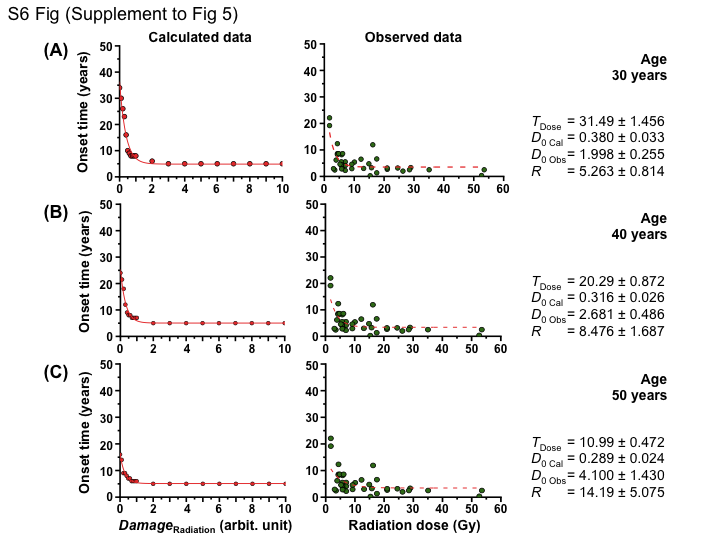

Supplement: S6 Fig — First, the calculated data in left panels were fitted to the following equation: T = TMin + TDose e–D/Do, where T, TMin, TDose, D and D0 are onset time (years), minimum onset time (years), dose-dependent onset time (years), DamageRadiation (in an arbitrary unit) and slope, respectively. Second, the Merriam and Focht data (right panels) were fitted to the same equation, but with the TDose value obtained from the calculated data and D indicating dose in Gy. Last, R was calculated as D0 for the Merriam and Focht data (D0 Obs) divided by D0 for the calculated data (D0 Cal), each for age 30, 40 and 50 years (panels A–C). The means and standard errors (SEs) of the yielded values for TDose, D0 Cal, D0 Obs and R are shown in each panel. For error bars in S6A–S6C Fig, see those in Fig 6A–6C. (TIFF) [file pone.0221579.s007.tiff]

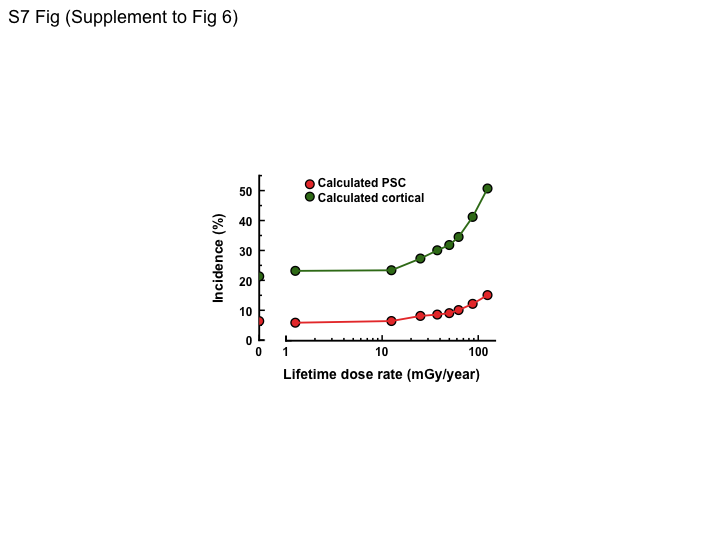

Supplement: S7 Fig — Exposures were assumed to occur at age 0–80 years at a constant dose rate of 1.25, 12.5, 25, 37.5, 50, 62.5, 87.5 and 125 mGy/year, respectively that gives a lifetime cumulative dose of 0.1, 1, 2, 3, 4, 5, 7 and 10 Gy at age 80 years. Red and green circles show the calculated data for posterior subcapsular (PSC) and cortical cataracts, respectively. (TIFF) [file pone.0221579.s008.tiff]
